# Diabetes Melitus

**Published:** 1861-07

**Authors:** Juriah Harriss

**Affiliations:** Prof. Physiology in Savannah Medical College, Savannah, Ga.


### SELECTIONS.

*Diabetes Mellitus—Its Pathology.* By Juriah Harriss, M. D., Prof. Physiology in Savannah Medical College, Savannah, Ga.

It is a fact that none will question that mankind owes an immense debt of gratitude to the medical men of the nineteenth century. With a few exceptions, the hiding-places of all diseases, their symptomatology and pathology, have been discovered and carefully marked out upon the map of science. The present generation of medical men has accomplished more than its quota in the prevention, cure, and in locating and noting the changes of tissues with the various and unlimited pathological phenomena which present themselves. The profession is laboriously endeavoring to elucidate the seat and phenomena of disease upon the only true and rational basis; first, to determine its location, the organs or tissues involved, then the nature of the modifications and variations from the natural or healthy structure. This accomplished, remedies can be easily and rationally applied, as preventive in the one, and curative in the other.

Diabetes is among the few diseases, the pathology and cure of which is not satisfactorily understood. To this, and a few others, the attention of the profession is now directed, and I believe with considerable plausibility of ultimate success. Like all diseases, the pathology of which is not clearly known, the the treatment is variable and unsatisfactory.

Zench, a German, first announced that saliva changed starch of bread, potates, beans, etc., into sugar, and some supposed the superabundance of this article in the system arose alone from this source.

McGregor next advanced the theory that the pathology of Diabetes was the peculiar action of *grastic juice* upon food. That this juice not only converted starchy matter of food into sugar, or glucose, but even meats were, by a perverted action of the gastric juice, changed into sugary materials. Thus, in his view, the food was the sole source of sugar in the economy.

Both of these theories have been proven untrue. The first utterly untenable; the latter embracing but a portion of the truth, as it ignores the principal source of glucose, or diabetic sugar. There are at present no adherents to the first opinion, that the sugar is derived by the action of saliva upon starch in food. It has been incontestably proven by experiment, in the labratory and the animal organism, that saliva produces no such change. The acidity of the gastric juice prevents the change, both in the test tube, when added to a solution of starch in saliva, and in the living animal when swallowed. It is unnecessary to say more upon this theory.

McGregor entertained the opinion that there were no anatomical lesions involved in this disease, but was purely a functional derangement of the stomach. That by this functional pervertion, all kinds of food, whether containing starch or not were converted by the gastric juice into sugar. Dr. Wood seems to indorse this opinion. He says: "It was ascertained by him (McGregor) that sugar is formed in the stomach of a diabetic patient during chymefication, even when no particle of saccharine matter has been swallowed. This happens to a certain extent, though the patient may have been confined to animal food exclusively.

Two individuals, one in health and the other diabetic, were vomited and purged, then fed on beef and water exclusively for three days, and at the end of this time were again vomited with sulphate zinc, administered about three hours after a meal. The matter thrown up by the healthy person showed no signs of fermentation; that of the diabetic patient fermented briskly, though less so than in another case, in which the diet was of the ordinary character."

"*Sugar, then, is produced in the stomach, at least of diabetic individuals, during the process of digestion.*"

Dr. Wood is not alone in the advocacy of this theory, and the error is therefore worthy of an expose.

McGregor found in the diabetic, sugar in the gastric juice, and mixed up with food, even when it contained no starchy matters. The observation was correct. But he found it in the saliva, the perspiration, urine, epithelial scales, etc., His conclusion would have been as logical if he had stated that the salivary glands, or skin, or kidneys, had performed the office. In this disease, all secretions, even the fluid of an ovarian cyst, contain sugar, as well as the contents of the stomach. Why? Simply because the blood is surcharged with sugar, and it passes from this circulating fluid, by the law of exosmosis, into all the secretions, without exception, as well as any other substance in solution in the blood, when in superabundance. Inject any substance in fluid form into the blood-vessels, to a considerable amount, and it will pass into all the secretions. The presence of sugar in the stomach during chymefication in a diabetic, is a fixed fact, but its existence in this organ does not prove that it is formed there. But more positive proof can be adduced to show the fallacy of this theory. Experiments, upon living animals, thanks to Bernard and others, are not only abundant, but conclusive, going to exhibit the error of this view of the pathology.

Early in the history of experimental physiology, Beaumont determined the fact that vegetables were not digested in the stomach. A little latter, the same fact was confirmed by a different process, frequently repeated. An individual, a short time after eating, was given an emetic, the meats were thrown up; the vegetables had, at an early hour, passed into the duodenum, as none of the latter was vomited. This was seen by Beaumont through the fistulous opening. Later still, Bernard and other experimental physiologists, have reaffirmed this statement. I have, by experiments, arrived at a similar conclusion. The gastric juice, or chymefication, has nothing to do with the digestion of non-agotized or starchy food. The experiments instituted are easy of performance, and can be executed by any who has a dog at hand. A fistulous opening is made into the stomach of a dog, and a silver canular introduced and secured by ligatures. Pure starch is given to the animal to eat. After a lapse of half hour or three quarters, the contents of stomach is allowed to pass through the canular, and collected in a vessel. This, submitted to analysis, shows the starch unaltered, no sugar formed. The same with bread, potatoes, etc. This proves that starchy food is not changed into sugar by the action of gastric juice, or during chymefication ; and the experiments previously referred to, makes it conclusive that the action of saliva upon any amylaceous food is checked by the gastric juice—so that in the economy no sugar is formed from this class of food, from the time it enters the mouth until it passes from the stomach into the duodenum.

McGregor's theory is then an empty hypothesis. Amylaceous aliments are digested, not in the stomach, but in the duodenum. The bile alone does not change or modify it. This may be proven by the admixture of bile with starch, in a test tube, with moderate heat. By a similar experiment with pancreatic juice, no change will be induced. But if bile and pancreatic juices are combined in a test tube with starchy matters, they will be changed into glucose. The digestion of this class of food, therefore, requires the combined action of these two digestive fluids. Neither acts upon them when uncombined. If cane-sugar is eaten, as in coffee, tea, syrups, etc., they are unchanged by saliva, or gastric juice, but the latter fluid makes them acid, which is *necessary* to the subsequent action of bile and pancreatic juice to change it into glucose. This may be also proven in a test tube. If cane sugar, or that of commerce, is disolved and added to bile and pancreatic juice, with temperature of animal heat, no change will occur. But if an acid, as hydrochloric, is added, to induce acidity, the cane sugar will soon be transformed into glucose. This is an exact imitation of what occurs in the econemy. Cane-sugar, of commerce, or in form of candy, is made acid when it reaches the stomach by the free acid of gastric juice, and then, only then, the bile and pancreatic juice is enabled to digest or change it into glucose. How absurd then the administration of sugar to cure its accumulation? It is well known that sugar and candy, eaten in large quantities, and frequently as happens through indulgent parents to children, that decayed teeth are the result. Why is this? The saliva acts upon the sugared particles retained in the mouth, by catching between the teeth, etc., Thus caught and retained, saliva has full opportunities to act unchecked, and lactic acid is found. This acts upon the imperfectly protected teeth, and hence their early decay. That portion of sugar or candy swallowed is not thus converted into lactric acid, because, as before stated, the transformation is prevented by the acidity of gastric juice.

It is clear then, that glucose, normally found in the system, and performing the important office, *in part*, of furnishing material for animal heat, is derived, to a certain extent, from amylaceous food, or that containing starch or cane sugar. But this is not the only source. If a healthy individual eats no cane sugar, nor food containing materials which can, in or out of the system, be changed into glucose, as an exclusive meat diet, glucose will still be found in the system. It must then be manufactured some where in the body. Bernard determined, incontrovertibly, by numerous experiments upon healthy animals, that sugar was always present in some portions of the circulation. To settle this point, and determine from whence it came, he instituted the following experiment, which has often been repeated by himself and others: Two dogs were taken; one was fed for some days, and even months, on amylaceous food; the other on meats. The jugular vein of each was opened, and a tube passed into the right auricle. The blood from each dog was analyzed, and sugar found in both—proof conclusive that sugar must have been formed in the system, *regardless* of the kind of food eaten. The next point in the experiment was to determine the origin of this material, or in what part of the system it was manufactured.

To have reached the right auricle, it must have gone through superior or inferior vera cava, or both. Blood analyzed from the superior contained no sugar, but discovered in the blood from the inferior cava. Sugar, then, normally found in circulation, must have been formed at a point below the heart. Blood, taken from the crural veins, contained none, nor did that in inferior cava, below the liver. It was then certain that sugar was not manufactured in the superior or inferior extremities, and its point of formation must by this process of exclusion, be derived from the abdomen. The experimental process of exclusion was continued a step further. The following experiment was performed: If a large opening was made through abdominal walls, and blood taken from vena porta, it was found to contain sugar, whether the animal fed on starchy food or meats. For the reason that the contents of hepatic veins regurgitated into vena porta, by absence of pressure of abdominal walls. But if a small opening was made, the vena porta contained no sugar, the pressure not being removed, provided the animal had been fed on meats. Should he have eaten amylaceous food, of course a small quantity would be present, because these materials would have been changed into glucose by the action of bile and pancreatic juice, and absorbed by the mesentine veins. The glucose then found in the vena cava inferior above the liver, in right side of the heart, even when the animal was confined to meats, must come from the liver. This was the manufacturing organ. It was invariably found in the tissue of the liver. Its presence renders the liver of animals eatable; deprived of this, it is bitter and nauseous to the taste.

From these experiments Bernard first came to the conclusion that glucose was a veritable *secretion* of the liver, and under the control of the pneumogastric nerve. But more recent and multiplied experiments, by himself and others, have induced him to modify his view, and he is sustained in the opinion, that the *liver does not secrete* glucose, but forms a *glucogenic matter*, which is transformed into glucose soon after entering the circulation, even in its own structure. Hence it is found in the substance of the liver, and in vena cava, after it receives blood from this organ. By what element of the blood the change is produced is not positively known.

We thus have sugar manufactured in the system in the physiological state. This leads us one step in advance, or towards solving the mysterious question of its superabundance in the system, called Diabetes. Subsequent experiments throw further light upon the pathology. Bernard discovered that glucogenic matter, after being formed by the liver, changed in the circulation into sugar, was also under the control of the pneumogastric nerve, and, though glucose was not a secretion of this organ, the glucogenic matter was. This fact was determined thus: If a pneumogastric nerve was cut in an animal, the amount of sugar was at once diminished. If irritated by pinching or scratching, sugar was increased. So with the root of the nerve. If the origin of pneumogastric was pricked with a pointed instrument, the same result followed—an increase of sugar in the system. Indeed, artificial Diabets could be induced at will, by irritating the origin of this nerve. Analyze the urine of a rabbit in a healthy state—no sugar will be found. Pass a needle between occiput and atlas, so directed as to prick the origin of the pneumogastric nerve, and sugar will soon make its appearance in the urine, and this in proportion to the amount of irritation induced. The experiment, however, to be successful, requires but a slight injury to the medulla oblongata, for if extensive the animal will die. As soon then as the irritation subsides, as when the nerve is irritated, the sugar in urine again disappears. *Diabetes Meliltus* may be produced at will, by irritation of the nerve controlling the formation of the glucogenic matter, by the liver. The disease thus produced is evidently a *neurosis*. So I believe it whenever it may occur. A functional derangement of an organ, due to nervous perversion, I am too much of a materialist, in pathology, to entertain the opinion that any disease is *purely* functional. With Broussais, I think every disease has a *local lesion*, not always inflammatory, as he taught, but often the contrary. It is true that some of these lesions are not determined, nor their location nor extent known, but must, nevertheless exist. From such source all pathological phenomena arise. Let us take as examples those which appear to be the most clearly functional diseases, epilepsy and hysteria. In many of these cases, *not all*, no pathological lesions can be discovered by the eye or aided by the microscope, and yet it is unscientific to conclude, because no lesion can be found, that none exists. In epilepsy often no change of encephalon or spinal cord can be discoved. In all neuroses there must be a change in the structure of the nerves or nervous centres, oftentimes molecular, unperceived by the eye, even with the aid of the microscope. In convulsive diseases, of reflex character, induced by irritants, indigestible matter in the intestines, teething of children, etc., the irritation causes a change in the molecules of the periperal extremities of the nerves, which change may cease when the irritation ceases, or may be extended to the nervous centre, and then subside or continue. To say there is no change, would be as wise as to conclude that there is no such thing as gravitation, because we see no mechanical agent at work. It is most unrational to entertain the opinion that epilectic convulsions can be produced by any other than structural or patholical changes in some nerve or nervous centre probably the *medulla oblongata*. The action of remedies, is another reason for entertaining the opinion that Diabetes Mellitus is a nervous disease. Any remedy, although directly opposed in character and therapeutic effects, modify the amount of sugar in the system, in this disease, provided it acts principally on the nervous system. If an alkali is given, the amount of sugar will be diminished, but as soon as the system becomes accustomed to it the remedy loses its effect. If then it is followed by an acid, the sugar will again diminish, but only for a short time, and vice versa. If an acute disease, as pneumonia, supervenes on an attack of Diabetes, the sugar will almost entirely disappear from the urine, to re-appear when the pneumonia is relieved. Like the remedies the inflammatory action perverts the nervous action, produces a more powerful impression on nervous system, and the other succumbs for the time. This can only occur in nervous diseases or nuroses.

In my view then Diabetes Mellitus *is caused by an irritation of pneumogastric nerve, at peripheral extremities, in stomach or liver, or along its course, or at its origin.* It is well known to physiologists that the same phenomena will follow, whether a nerve is irritated at the extremities, along the course of the nerve, or the nervous centre, at its point of origin. An irritation of any of these three points of this nerve, produces an increased activity on the part of the liver in forming the glucogenic matter, which is as much a secretion of this organ as the bile. The amount of glucogenic matter thus secreted, and afterwards in the circulation, changed into sugar, is more than can be destroyed in the lungs, and accumulates in the system. The blood becomes highly charged with it, and it passes from the circulation with gastric juice, bile, perspiration, and urine. Most abundantly in the urine, because this is the most active secretion in the body.

In this disease amylaceous food should be avoided, for the reason already indicated, that this class of aliments is changed during the process of digestion into glucose. The plain indication is to cut off this source of sugar, when the system is too abundantly supplied with this material. The second therapeutic indication is to relieve the irritation, whether at the peripheral extremities, in the liver, along the course of the nerve, or at its point of origin. That this nerve is at fault, I think conclusive, but at which point the irritation most usually exists, is yet doubtful. I incline to the opinion that generally the seat of irritation is either the medulla oblongata, or the peripheral extremities, distributed in the substance of the liver. Hence our remedies should be directed to either one or the other of these points.

Pepsine, sugar, etc., as curative agents in this disease, are worse than useless.—*Savannah Journal of Medicine.*

